# Spindle Orientation-Independent Control of Cell Fate Determination by RGS3 and KIF20A

**DOI:** 10.1093/texcom/tgaa003

**Published:** 2020-02-20

**Authors:** Runxiang Qiu, Kiyohito Murai, Qiang Lu

**Affiliations:** 1 Department of Developmental and Stem Cell Biology, Beckman Research Institute of the City of Hope, Duarte, CA 91010, USA; 2 Department of Macroscopic Anatomy, Nagasaki University Graduate School of Biomedical Sciences, 1-12-4 Sakamoto, Nagasaki 852-8523, Japan

**Keywords:** cell cleavage plane, cell fate determination, cerebral cortex, KIF20A, mitotic spindle orientation, neural progenitor cells, proliferation versus differentiation, RGS3

## Abstract

It was proposed that similar to its role in the invertebrate nervous system, mitotic spindle orientation (or cell cleavage plane orientation) of a dividing neural progenitor cell specifies the fate of daughter cells in the mammalian brain, modulating the production of neurons via symmetric versus asymmetric cell divisions during the course of neurogenesis. Experimental tests of the sufficiency of spindle/cleavage plane orientation in mammalian cell fate determination have yielded conflicting results. On the other hand, the necessity of spindle/cleavage plane orientation in mammalian cell fate determination has not yet been addressed. Here we examined the necessity of spindle/cleavage plane orientation during cortical neurogenesis in mice with loss-of-function of the RGS3-KIF20A interaction axis. We present evidence that while inactivation of RGS3 or KIF20A was linked to a shift in neural progenitor cells from proliferative to differentiative divisions in the developing cortex, these genetic mutations did not lead to anticipated alteration in the orientation of spindle/cleavage plane. Our results indicate that the RGS3-KIF20A axis regulates the balance between proliferation and differentiation in the mammalian cortex employing a mechanism independent of spindle/cleavage plane orientation. These data also caution against using spindle/cleavage plane orientation as the synonym for cell fate determination.

## Introduction

Radial glial cells (RGCs), the primary neural progenitor cells (NPCs) of the mammalian cerebral cortex, are highly polarized cells with the nucleus located in the ventricular zone (VZ) and a long radial process connecting to the pial surface. During development, RGCs first proliferate to expand their population and later progressively enter asymmetric cell divisions to sequentially generate neurons and glia. Given the similar polarized nature of invertebrate NPCs and the importance of mitotic spindle orientation, or more precisely cell cleavage plane orientation, in asymmetric cell division in the invertebrate nervous system (Homem and Knoblich 2012; Homem et al. 2015; Venkei and Yamashita 2018), a causal link between spindle/cleavage plane orientation of mitotic RGCs and daughter cell phenotype in the mammalian brains was suggested, based on the observation of correlations between spindle/cleavage plane orientation and asymmetric inheritance during cortical neurogenesis (Chenn and McConnell 1995). The mitotic spindle orientation model hypothesized that horizontal divisions (cleavage planes perpendicular to the ventricular surface) are preferentially symmetric progenitor self-renewals, while vertical divisions (cleavage planes parallel to the ventricular surface) tend to be neurogenic. It was later revised that neurogenic divisions include both oblique and vertical divisions (Kosodo et al. 2004), as strictly vertical divisions are rare in the mammalian cortices. The mitotic spindle orientation model predicted that as cortical development progresses from the early phase of progenitor expansion to the phase of active neurogenesis, spindle/cleavage plane orientations correspondingly shift from preferentially horizontal divisions to more oblique and vertical divisions, causing cleavage furrow bypassing the apical membrane domains that may harbor key cell fate determinants. However, analyses of spindle/cleavage plane orientations in dividing RGCs at neurogenic stages of cortical development obtained data inconsistent with the hypothesis. For examples, analyses of angles of divisions in the developing mouse and rat cortices have found that RGCs at the ventricle mainly take the form of horizontal divisions (Stricker et al. 2006; Konno et al. 2008; Noctor et al. 2008), an observation that is difficult to reconcile with massive neuron production during the period of cortical neurogenesis. Time-lapse imaging of cortical slice cultures revealed complex rotation and oscillation of the mitotic spindle before progenitor cell division; however, the data also found that cleavage orientation was not a valid predictor of cell phenotype as disparate daughters can be generated by the same spindle behavior and orientation (Haydar et al. 2003). Time-lapse imaging studies also indicated that the majority of asymmetric RGC divisions segregate the apical membranes to both daughter cells (Matsuzaki and Shitamukai 2015; Delaunay et al. 2017), an observation inconsistent with segregation of the apical cell fate determinants hypothesized by mitotic spindle orientation model. In addition, experimental exploration of the mitotic spindle orientation model by modulating the activity of some key polarity-related factors identified in the invertebrate studies have so far yielded conflicting results in the mouse cortex. For instances, Gβγ complex, but not Gαi subunit, of the heterotrimeric G proteins was reported to regulate cell fate in cortical NPCs based on mitotic spindle orientation analyses (Sanada and Tsai 2005); however, our genetic studies of mice carrying a constitutively active Gαi subunit revealed that Gαi subunits are strong initiators of neuronal fate in daughter cells (Murai et al. 2010). Modulation of the activity of LGN/GSPM2, a modulator of G protein signaling, caused randomization of mitotic spindle orientation (Konno et al. 2008) and affected the rate of outer radial glia generation (Shitamukai et al. 2011), a phenomenon also observed in the study of human NPCs (LaMonica et al. 2013), but did not induce a switch from symmetric self-renewal divisions to asymmetric neurogenic divisions (Konno et al. 2008). Other experiments of perturbing microtubule- or spindle-regulating factors have also reported divergent results with respect to a possible role of spindle/cleavage plane orientation in cell fate determination (Fish et al. 2006; Yingling et al. 2008; Gauthier-Fisher et al. 2009; Postiglione et al. 2011; Mora-Bermudez et al. 2014). Most importantly, however, while all studies to date have investigated whether spindle/cleavage plane orientation may influence the mode of cell division during mammalian neurogenesis, the flip side of the question, i.e., whether the orientation of spindle/cleavage plane is required for controlling proliferative versus neurogenic cell divisions in cell fate specification remains untested.

We have found that the EphrinB-RGS3-KIF20A axis of protein–protein interaction network and the Gα subunit work together to regulate the balance between proliferation and differentiation in the developing mouse cortex. Genetic inactivation of Ephrin-B1 (Qiu et al. 2008), PDZ-RGS3 (RGS3 isoform 1) (Qiu et al. 2010), mitotic kinesin KIF20A (Geng et al. 2018), or genetic activation of Gα subunit (knockin of an RGS-insensitive Gαi2 mutant, G184SGαi2) (Murai et al. 2010) causes cortical NPCs to leave the cell cycle, leading to a shift of the balance in RGCs from proliferation to neuronal differentiation. We reasoned that these strains of mutant mice would present a suitable genetic platform to test the role of spindle/cleavage plane orientation in cell fate determination. Should spindle/cleavage plane orientation be functionally essential for cell fate choice, i.e., symmetric proliferative division versus asymmetric neurogenenic division, these mice would reveal changes in orientations in favor of neuronal differentiation. Specifically, these mice are expected to show a switch in progenitor cells from horizontal divisions (progenitor expansion) to vertical or oblique divisions (neuronal differentiation). In this report, we present our analyses of the in vivo patterns of cell cleavage planes in dividing RGCs of the *Pdz-Rgs3* and *Kif20a* knockout mice. Our data found no detectable connection between the cell fate-regulating function of RGS3 or KIF20A and cleavage plane orientation.

## Materials and Methods

### Embryonic Brain Sample Preparation

Animal procedures were approved by the Institutional Animal Care and Use Committee. Preparation of brain samples from the littermate wild-type and mutant mice was previously described (Qiu et al. 2010; Geng et al. 2018). Briefly, wild-type and mutant embryonic brains were dissected out at E11.5 or E15.5, fixed in 4% paraformaldehyde at 4°C overnight, cryoprotected in 30% sucrose, and embedded in OCT. Brain sections of 12 μm thickness were obtained, stained with Hoechst 33258 (1 μg/ml), and mounted with fluoromount G. Sections used in this study were from the same brain samples used in phenotype analyses in the previous two studies (Qiu et al. 2010; Geng et al. 2018). Brain images were taken using a confocal microscope (Zeiss LSM 510 Upright 2 photon) at ×40 optical view.

### Cell Cleavage Plane Analysis

Apically localized dividing progenitor cells at Anaphase/Telophase were identified with the appearance of typically condensed separating chromatids. Angles of cleavage planes with reference to the surface of the VZ were determined using Image-Pro Premier (Media Cybernetics, Inc). The data of each individual brain were obtained from 30 to 50 pictures of brain sections at ×40 optical view.

### Statistical Analysis

Kolmogorov–Smirnov (K–S) test was used to compare distribution of cell cleavage plane angles between mutant and wild-type brains from the *Rgs3* and *Kif20a* knockout mice. No statistically significant differences between mutant and wild-type divisions in the three data groups could be detected.

**Figure 1:**
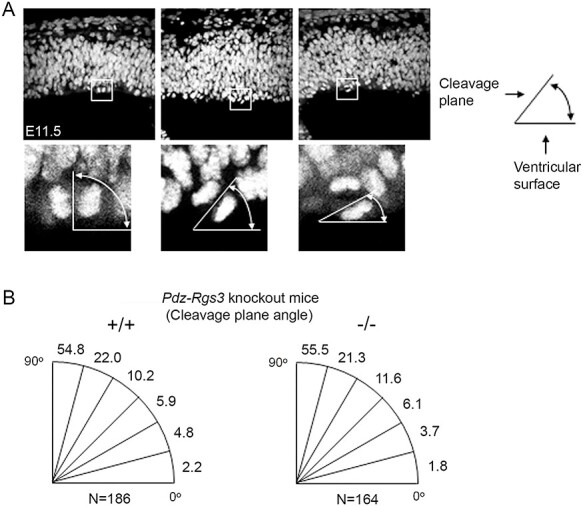
Analysis of cell cleavage plane orientation in E11.5 cortex of the *Pdz-Rgs3* germline knockout mice. (*A*) Examples of Hoechst-stained anaphase/telophase chromatids with various cleavage angles with reference to the ventricular surface. Dividing apical cells—cells that are located at the ventricular surface—were included for analyses. (*B*) The graphs show distributions of cleavage plane angles at 15° intervals in brains of the wild-type and *Pdz-Rgs3* germline knockout mice. Numbers indicated percentages that were calculated based on combined data of each genotype group summarized in Table 1*B*. K–S test showed that there was no significant difference in distributions of cleavage plane angles between mutant and wild-type brains.

## Results

**Figure 2:**
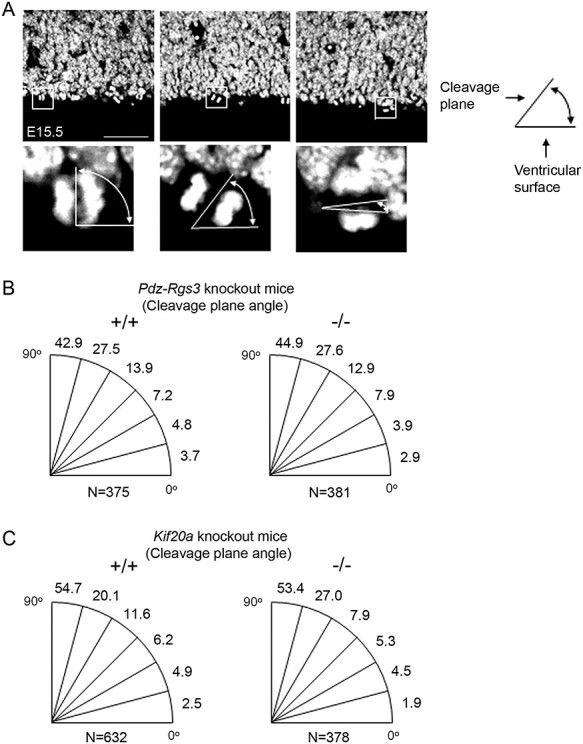
Analysis of cell cleavage plane orientation in E15.5 cortex of the *Pdz-Rgs3* and *Kif20a* germline knockout mice. (*A*) Examples of apical cell divisions with various angles in the E15.5 cortices. Scale bar represents 100 μm. (*B*) The graphs show distributions of cleavage plane angles at 15° intervals in brains of the wild-type and *Pdz-Rgs3* knockout mice (percentages were based on Table 2*B*). (*C*) The graphs show cleavage plane angle distributions in brains of the wild-type and *Kif20a* knockout mice (percentages were based on Table 3*B*). K–S test showed that there were no significant differences in distributions of cleavage plane angles between mutant *Rgs3* or *Kif20a* and wild-type brains.

We began by examining the planes of division in cortical NPCs of the *Pdz-Rgs3* germline knockout mice at embryonic day 11.5 (E11.5), an early stage of progenitor cell expansion. As the cortex contains various types of progenitor cells, including RGCs, outer subventricular zone progenitor cells (Fietz et al. 2010; Hansen et al. 2010; Wang et al. 2011), intermediate progenitor cells (IPC) or basal progenitor cells (Haubensak et al. 2004; Miyata et al. 2004; Noctor et al. 2004), and short neural progenitor cells (Tyler and Haydar 2013), we focused our analyses on dividing RGCs located at the apical surface. To investigate the in vivo patterns of cell cleavage plane distribution, we stained sections of E11.5 brains with nuclear dye and documented confocal images. To maintain consistency in analyses, we focused on dividing progenitor cells with the following two characteristics: 1) being localized at the apical surface of the VZ (for scoring apical RGCs) and 2) showing condensed separating chromatids (for conspicuously identifying dividing cells at Anaphase/Telophase of mitosis). The angles of the cleavage planes were determined with reference to the apical surface of the VZ (Fig. 1*A*). Consistent with what was observed in previous studies (Stricker et al. 2006; Konno et al. 2008; Noctor et al. 2008), most of the occurring cell divisions were horizontal divisions in the cortices of the wild-type brains (Table 1 and Fig. 1*B*). Among littermates of the *Pdz-Rgs3* knockout mice, homozygous mutant brains showed an overall comparable distribution of cleavage orientations compared with wild-type brains and there was no noticeable shift of cleavage plane orientations from the predominant horizontal divisions to more oblique and vertical divisions at this developmental stage. We next examined the mutant brains of the *Pdz-Rgs3* knockout mice at E15.5 (Fig. 2*A*), a peak stage of cortical neurogenesis when we previously observed an early cell cycle exit and precocious neuronal differentiation in these mutant brains (Qiu et al. 2010). Our data showed that distributions of cleavage plane orientations among littermate brains of E15.5 were also largely comparable with no obvious deviation from the predominant feature of the occurring horizontal cell divisions (Table 2 and Fig. 2*B*).

**Table 1:** Analysis of cleavage planes of apical mitotic cells in the E11.5 cortices of the *Pdz-Rgs3* germline knockout mice. (*A*) Summarizes the analysis of cleavage plane distribution in 15° intervals in each individual littermate brains (three wild-type and three homozygous mutant brains). The data of each brain represent the analyses of anaphase/telophase cells from 30–50 confocal pictures of ×40 optical view (examples are shown in Figure 1A). (*B*) is the combined data from (*A*) showing the cleave plane distributions in wild-type or mutant brains

A						
Angles	Genotypes					
	+/+			−/−		
	#1	#2	#3	#1	#2	#3
0–15	1	1	2	1	2	0
15–30	4	3	2	3	3	0
30–45	2	3	6	4	4	2
45–60	5	5	9	9	5	5
60–75	16	14	11	14	6	15
75–90	37	31	34	46	18	27
B						
Angles	Genotypes			
	+/+	−/−				
0–15	4	3				
15–30	9	6				
30–45	11	10				
45–60	19	19				
60–75	41	35				
75–90	102	91				

To further evaluate cleavage plane orientation in relation to cell fate determination, we next analyzed cleavage planes in mice with germline knockout of the *Kif20a* gene. Our previous analyses of homozygous mutant brains and their wild-type littermates showed that knockout of *Kif20a* resulted in early cell cycle exit and precocious neuronal differentiation of cortical NPCs (Geng et al. 2018). Examination of cleavage plane orientations between these mutant and wild-type littermate brains showed that the predominant pattern of horizontal divisions observed during normal cortical neurogenesis was also preserved in the homozygous *Kif20a* mutant brains (Table 3 and Fig. 2*C*).

**Table 2:** Analysis of cleavage planes of apical mitotic cells in the E15.5 cortices of the *Pdz-Rgs3* germline knockout mice. (*A*) Summarizes the analysis of cleavage plane distribution in 15° intervals in individual littermate brains (three wild-type and three homozygous mutant brains). (*B*) is the combined data from (*A*) showing the cleave plane distributions in wild-type or mutant brains

A						
Angles	Genotypes					
	+/+			−/−		
	#1	#2	#3	#1	#2	#3
0–15	5	8	1	4	4	3
15–30	9	9	0	6	5	4
30–45	12	12	3	11	9	10
45–60	21	23	8	15	19	15
60–75	44	51	8	33	39	33
75–90	71	66	24	61	55	55
B						
Angles	Genotypes			
	+/+	−/−				
0–15	14	11				
15–30	18	15				
30–45	27	30				
45–60	52	49				
60–75	103	105				
75–90	161	171				

**Table 3:** Analysis of cleavage planes of apical mitotic cells in the E15.5 cortices of the *Kif20a* knockout mice. (*A*) Summarizes the analysis of cleavage plane distribution in 15° intervals in individual littermate brains (four wild-type and four homozygous mutant brains). (*B*) is the combined data from (*A*) showing the cleave plane distributions in wild-type or mutant brains

A								
Angles	Genotypes							
	+/+				−/−			
	#1	#2	#3	#4	#1	#2	#3	#4
0–15	4	3	6	3	2	4	1	0
15–30	9	6	9	7	6	5	3	3
30–45	12	7	15	5	11	5	1	3
45–60	18	7	33	15	10	12	2	6
60–75	34	26	45	22	25	59	4	14
75–90	123	51	112	60	54	111	19	18
B								
Angles	Genotypes			
	+/+	−/−				
0–15	16	7						
15–30	31	17						
30–45	39	20						
45–60	73	30						
60–75	127	102						
75–90	346	202						

Together, these results showed that there were no discernible differences in cell cleavage plane orientations of cortical NPCs in these two strains of knockout mice, although RGS3 and KIF20A were both crucial for cell fate determination during cortical neurogenesis. These data thus indicated that the observed cell fate changes in the brains of the *Pdz-Rgs3* or *Kif20a* knockout mice were not linked to changes of cleavage plane/spindle orientations in apically localized mitotic RGCs.

## Discussion

Initially formulated to explain neuronal differentiation process in the *Drosophila* neuroblast system, the mitotic spindle orientation hypothesis presented an intriguing model for understanding how symmetric versus asymmetric cell divisions might be balanced during development (Homem and Knoblich 2012; Homem et al. 2015; Venkei and Yamashita 2018). During the past two decades, the notion of a causal link between spindle/cleavage plane orientation and cell fate determination has been extended into studies of the mammalian brain and was thought to be a major mechanism governing the control of proliferation versus differentiation during mammalian neurogenesis (Homem et al. 2015). More recently, the same concept was also introduced to several other stem/progenitor cell systems with epithelial or polarized characteristics (e.g., Kulukian and Fuchs 2013; Williams and Fuchs 2013). Furthermore, spindle/cleavage plane orientation defects were proposed to contribute to brain malformation and some neurological disorders, such as microcephaly and lissencephaly, or tumorigenesis, as a result of postulated imbalance of proliferation versus differentiation during stem/progenitor cell development. However, to date, conclusive evidence demonstrating the sufficiency of spindle/cleavage plane orientation for cell fate determination concerning the control of proliferation versus differentiation in the mammalian brain is still lacking (Peyre and Morin 2012; Shitamukai and Matsuzaki 2012; Matsuzaki and Shitamukai 2015; Delaunay et al. 2017) and a direct causal role of spindle/cleavage plane orientation in the proposed brain diseases remains to be established (Noatynska et al. 2012).

In this study, we have investigated the mitotic spindle orientation model from the perspective of necessity, i.e., whether spindle/cleavage plane orientation is required for fate decision of proliferation versus differentiation. We examined distributions of cell cleavage plane orientations in two strains of germline mutant mice that displayed a fate change in daughter cells during cortical neurogenesis. Among different subtypes of progenitor cells within the cortical VZ, we focused our analysis on dividing RGCs apically localized. We previously found that RGCs produce most, if not all, projection neurons through IPCs (Liu et al. 2016), which are committed to becoming neurons, and thus the cell fate function of RGS3 or KIF20A most likely occurs in the RGC divisions. Our data revealed that inactivation of the RGS3-KIF20A function did not lead to any obvious changes in the orientations of spindle/cleavage plane in dividing apical RGCs, even though *Pdz-Rgs3* or *Kif20a* knockout could cause these progenitor cells to switch from proliferative to differentiative divisions (Qiu et al. 2010; Geng et al. 2018). These data thus indicate that while mitotic spindle is critically important for ensuring proper progression of mitosis during cell division, orientation of the spindle or the cell cleavage plane is not essential for determining the outcome of proliferative or differentiative fate of the daughter cells in mammalian cortical neurogenesis.

An immediate question would be how a spindle/cleavage plane orientation-independent mechanism of RGS3 or KIF20A might work to regulate cell fate determination during cortical neurogenesis? We previously observed that during NPC divisions, RGS3, KIF20A, and Gα subunit, are all present in the intercellular bridge (ICB) (Geng et al. 2018), a dynamic membrane structure that is formed during cytokinesis and keeps the two nascent daughter cells attached before they are finally separated. Most recently, we further identified SEPT7 as a cell fate regulator that works together with the RGS3-KIF20A complex (Qiu *et al*. 2019). We speculate that KIF20A-associated complexes within the ICB carry some key pro-proliferative fate determinants, whose symmetric or asymmetric distribution or inheritance by daughter cells during cell abscission can result in specific fate commitment. Although the precise details of how KIF20A-associated complexes are segregated into nascent daughter cells is not clear, SEPT7-mediated membrane or cytoskeleton organization and remodeling process at or near the midbody within the ICB may play a crucial role. Importantly, this ICB-based process does not seem to have to rely on the orientation of spindle/cleavage plane for retaining symmetry or creating asymmetry in the separating daughter cells.

In summary, while the precise mechanism by which the RGS3-KIF20A-SEPT7 protein–protein interaction axis regulates the cell fate specification process during cytokinesis of mitotic RGCs requires further investigation, our current data indicate that fluctuation of spindle/cleavage plane orientation is not a requirement for the control of cell division mode in the mammalian brain. Importantly, our data cast a cautionary note on causally linking spindle/cleavage plane orientation to determination of cell fate.

## Notes

We thank Jeremy LaDou and animal core staff for assistance with animal breeding and care; Brian Armstrong and staff for the use of imaging facility. This work is supported by National Institutes of Health. *Conflict of Interest*: None declared.

## Funding

National Institutes of Health (grant NS096130 to Q.L.).
